# Supplementary material for: Exploring genetic signatures of zoonotic influenza A virus at the swine–human interface with phylogenetic and ancestral sequence reconstruction
Source: Virus Evol. 2025 Apr 26;11(1):veaf028. doi: 10.1093/ve/veaf028 (PMC12248185; doi:10.1093/ve/veaf028)
Supplement: veaf028_Supp [file veaf028_supp.zip › gisaid_supplemental_table_epi_set_250117dt.pdf]

SUPPLEMENTAL TABLE

Data Availability

GISAID Identifier:	EPI_SET_250117dt
doi:	10.55876/gis8.250117dt

All genome sequences and associated metadata in this dataset are published in GISAID's EpiFlu database. To view the contributors of each individual sequence with details such as accession number, Virus name, Collection date, Originating Lab and Submitting Lab and the list of Authors, visit 10.55876/gis8.250117dt

Data Snapshot

- EPI_SET_250117dt is composed of 257944 individual viruses;
- The collection dates range from 1479-08-26 to 2023-01-25;
- Data were collected in 191 countries and territories.
